# Functional Changes in Patients and Morphological Changes in the Lumbar Intervertebral Disc after Applying Lordotic Curve-Controlled Traction: A Double-Blind Randomized Controlled Study

**DOI:** 10.3390/medicina56010004

**Published:** 2019-12-19

**Authors:** Chang-Hyung Lee, Sung Jin Heo, So Hyun Park, Hee Seok Jeong, Soo-Yeon Kim

**Affiliations:** 1 Rehabilitation Medicine, Pusan National University School of Medicine and Research Institute for Convergence of Biomedical Science and Technology, Pusan National University Yangsan Hospital, Yangsan 50612, Korea; aarondoctor@gmail.com; 2 Research Institute for Convergence of Biomedical Science and Technology, Pusan National University Yangsan Hospital, Yangsan 50612, Korea; 3 Department of Physical Therapy, Youngsan University, Yangsan 50510, Korea; ptpsh@ysu.ac.kr; 4 Radiology Medicine, Pusan National University School of Medicine and Research Institute for Convergence of Biomedical Science and Technology, Pusan National University Yangsan Hospital, Yangsan 50612, Korea; mediknight@hanmail.net

**Keywords:** traction, lumbar lordosis, intervertebral disc, pain, function, morphology

## Abstract

*Background and Objectives:* Lumbar traction is widely used as a non-operative treatment for lumbar intervertebral disc disease. The effect of traditional traction (TT) using linear-type traction devices remains controversial for various reasons, including technical limitations. Thus, the purpose of this study was to compare the effects of the newly developed lumbar lordotic curve-controlled traction (L-LCCT) and TT on functional changes in patients and morphological changes in the vertebral disc. *Materials and Methods:* A total of 40 patients with lumbar intervertebral disc disease at the L4/5 or L5/S1 level as confirmed by magnetic resonance imaging were recruited and divided into two groups (L-LCCT or TT). The comprehensive health status changes of the patients were recorded using pain and functional scores (the visual analogue scale, the Oswestry Disability Index, and the Roland–Morris Disability Questionnaire) and morphological changes (in the lumbar central canal area) before and after traction treatment. *Results:* Pain scores were significantly decreased after traction in both groups (*p* < 0.05). However, functional scores and morphological changes improved significantly after treatment in the L-LCCT group only (*p* < 0.05). *Conclusions:* We suggest that L-LCCT is a viable option for resolving the technical limitations of TT by maintaining the lumbar lordotic curve in patients with lumbar intervertebral disc disease.

## 1. Introduction

Lumbar traction is widely used on patients with intervertebral disc disease, as a way to reduce pressure on the vertebral foramen by releasing tension between adjacent spinal vertebrae [1,2,3]. Thus, it can improve spinal alignment and reduce unnecessary muscle spasms surrounding the lesioned area [4]. In addition, the reduction of intervertebral pressure and restoration of the original lumbar lordotic curve can allow the protruded nucleus pulposus to be retracted inward [5]. However, previous studies have not clearly established the treatment effects of traction treatment. Some studies using pain and functional tests such as the visual analogue scale (VAS), the Oswestry Disability Index (ODI), the Roland–Morris Disability Questionnaire (RM), and 36-item Short Form Health Survey Questionnaire (SF-36) scores have shown significant improvements in traction patients compared to traditional physical therapy patients [6,7,8,9,10]. However, in other studies on patients with intervertebral disc disease, traction showed no significant benefits compared to other treatment techniques such as physical therapy and medication [11,12,13,14]. In spite of the theoretical benefits of traction treatment in relieving disc compression for patients with intervertebral disc disease, the clinical outcomes have not been very pronounced.

Since individual physical conditions vary, standard therapeutic guidelines need to be customized, which requires time and effort. As there are no standard protocols or previous well-designed studies for traction treatment, it may be difficult to evaluate the efficacy of traction treatment compared to a control. As suggested by Clarke et al. [13], it is difficult to empirically determine the effects of traction on patients with intervertebral disc disease due to difficulties in setting up initial conditions, conducting blind tests with a mechanical traction stress, and differences in patient education levels and understanding of disease mechanisms. Finally, there may be other conditions that cause the same symptoms, or different symptoms may arise from the same cause.

In addition to the reasons mentioned above, there can be technical limitations to applying traction in patients with intervertebral disc disease. One possible reason for a technical limitation in applying traction is the change in the lordotic curve. First, the lordotic curve may be decreased during lumbar traditional traction (TT). When traction force is applied to the spine in the TT method, the main effect is to straighten spinal structures, rather than decompress intervertebral discs. This also decreases the natural lordotic curve, and may be the cause of discomfort and poor treatment outcomes after spinal traction [14,15,16]. When the lordotic curve is decreased, posterior spinal structures, facet joints, and posterior longitudinal and interspinous ligaments are elongated more than anterior spinal structures. If this occurs during TT, it causes improper pressure loading on disc structures. Thus, excessively stretched posterior column structures might cause pain. Second, the position of the subject can influence spinal traction results. In supine positions, the lordotic curve decreases due to the vertical force of gravity on the lumbar curve, and this may decrease the lordotic curve more in patients with low back pain. When traction force is applied to the spine in a supine position using a TT method, the force of gravity affects the whole spine structure. Consequently, the lordotic curve is not restored, and this can cause pain.

Consequently, we wondered if applying a traction force to vertebrae while maintaining the lordotic curve would result in equal force distribution to the anterior and posterior parts of the spinal structure. In this sense, the lumbar lordotic curve-controlled traction device (L-LCCT), which targets the L3–5 intervertebral disc space, has been invented. In our previous study [17], we measured the morphological changes of the cervical intervertebral discs by applying L-LCCT and compared these findings with TT. We observed that the anterior and posterior spinal structures were equally elongated in the L-LCCT group, but disproportionally increased in the posterior section in the TT group. The improperly applied decompression force is regarded as a crucial factor in the poor outcomes observed in the TT group. In the L-LCCT group, the equally elongated anterior and posterior structures showed favorable pain, functional, and morphological changes.

To date, no study has measured functional outcomes and morphological changes of vertebral discs in lumbar intervertebral disc disease patients using L-LCCT. Therefore, the purpose of our study was to investigate the clinical efficacy of L-LCCT compared to TT in patients with lumbar intervertebral disc disease.

## 2. Materials and Methods

This study was a double-blinded randomized controlled trial comparing L-LCCT and TT. After acquiring informed consent, all patients with intervertebral disc disease were selected from the outpatient clinic. This study was conducted in accordance with the Declaration of Helsinki. All patients received sufficient explanation regarding the objectives and methods of the study before participating. Patients were randomly assigned into the two groups (L-LCCT or TT) by a research physician, with the help of a computer-generated table of random numbers. No patients knew which group they belonged to. Evaluations were carried out by a physician who was blinded to the treatment. This study was approved on 20 April 2016 by the institutional review board (IRB 04-2016-029).

### 2.1. Study Population and Sample

A total of 40 patients (17 male and 23 female) with lumbar intervertebral disc disease at the L4/5 or L5/S1 level, as confirmed by MRI (Magnetic Resonance Imaging), were recruited between June 2016 and February 2017. The inclusion criterion was a diagnosis of herniated intervertebral disc disease (HIVD) in the lumbar spine, with unrelenting low back pain and/or sciatica symptoms lasting more than 3 months. Exclusion criteria were as follows: patients with acute inflammation, unstable lumbar vertebrae, joint hypermobility, inhibited flexion or extension of the lumbar vertebrae, or a released disc fragment [18]. Each patient’s exercise activity was limited to daily activities not exceeding 6 metabolic equivalents (MET) (equivalent to 3.5 to 7 kcal/min; similar to walking for pleasure, or mild to moderate housework) [19]. During patients’ clinical interviews, information on all life activities was recorded. Those who did not keep these guidelines were excluded from this study.

### 2.2. L-LCCT Versus TT

**Figure 1:**
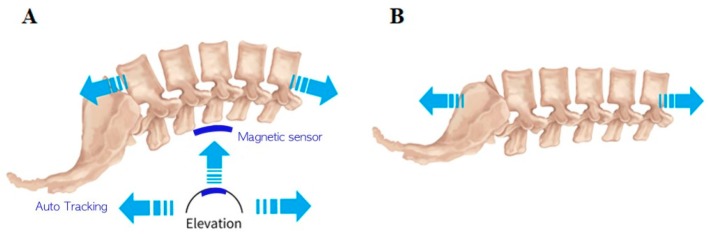
Lumbar traction techniques: (**A**) lumbar lordotic curve-controlled traction (L-LCCT) and (**B**) traditional traction (TT). A magnetic marker was attached to the skin at the L4 intervertebral disc space using physical palpation and an automated tracking system.

The L-LCCT (Kinetrac-9900, Hanmed Co., Gimhae, Korea) was used to maintain the natural lordotic curve of the spine by supporting the lumbar curve at the L3–5 intervertebral disc space. After the patient assumed a supine position, the chest and pelvis were belted. Initially, a magnetic marker was attached to the skin at the L4 intervertebral disc space by physical palpation and an automated tracking system (Figure 1). The automated tracking system ensured a lumbar lordotic curve during L-LCCT by elevating L3–5. A magnetic surface marker was attached to the patient’s L4 area, where the lordotic curve is in maximum. As the highest lordotic point moved during traction, the auto-tracking system followed the magnetic surface marker, and thus constantly maintained the lordotic curve. During L-LCCT, the height of the elevated lumbar lordotic curve support was increased to the most comfortable point for each patient. The operating range of the device did not exceed the human body’s range of motion. In addition, its maximum traction power did not exceed 100 pounds, or 50% of the patient’s weight, which prevented damage to the patient’s muscles and tendons.

The TT method was applied to patients without supporting the lumbar lordotic curve. We followed the same protocol as for L-LCCT, except without the lordotic curve modification, and with the patient lying in a supine position. According to previous reports and patient clinical compliance, all patients received traction three days per week for five weeks [3]. Traction duration was 15 min, and a total of 15 traction sessions were applied to each group.

### 2.3. Outcome Measurement using Pain and Functional Status

Pain and functional outcomes were measured before the first intervention and after the last treatment session.

Pain in the trunk and lower extremities that was exacerbated during daily living activities was measured using the mean pain score from the VAS, which ranges from 0 to 10. The patients were asked to place a mark along the line to denote their pain level, where 0 reflected “no pain” and 10 reflected the “worst pain”.

The ODI and RM were used to assess patients’ functional status. The ODI consists of 10 items that refer to daily living activities that might be disrupted by pain. These items are as follows: pain intensity, personal care, lifting, walking, sitting, standing, sleeping, sex life, social life, and traveling. Each item is rated on a six-point Likert scale. The total score is then translated to a scale ranging from 0 to 100, where 0 indicates no disability and 100 indicates the worst possible disability [20]. The RM is a 24-item reported outcome measure that inquires about disabilities resulting from lower back pain. Items are scored 0 if left blank or 1 if endorsed, for a total RM score ranging from 0 to 24—higher scores represent higher levels of pain-related disability [21]. Typical RM test–retest estimates are in the range of 0.79 to 0.88 points for relative reliability (intra-class correlation) and 1.7 to 2.0 points for absolute reliability [20].

### 2.4. Morphological Changes Measurement in Lumbar Disc Severity Using Lumbar MRI

**Figure 2:**
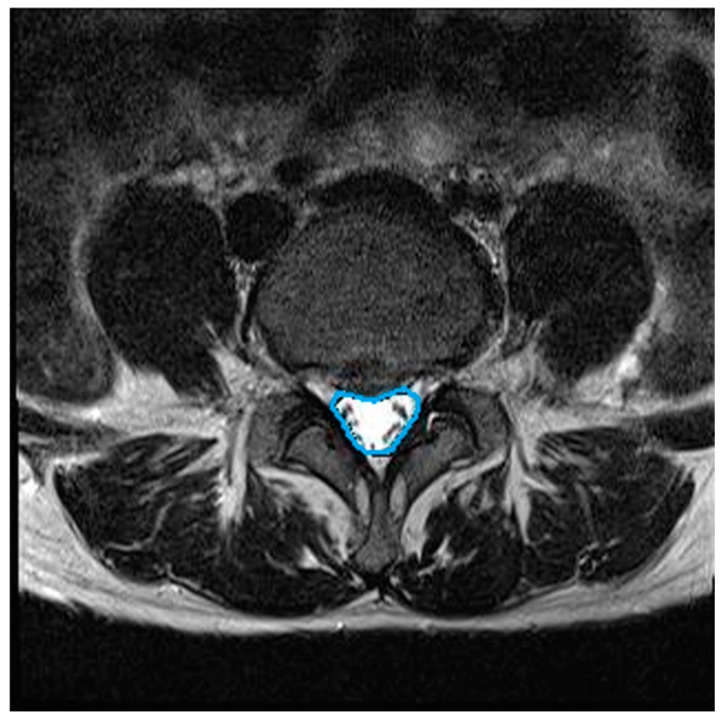
Central canal area in axial view of lumbar spine MRI (Magnetic Resonance Imaging).

We assessed the changes in the lumbar central canal area before and after lumbar traction. All MRI examinations were performed using a 3 Tesla MRI (Siemens Healthcare GmbH, Erlangen, Germany). Using T2-weighted images (field of view (FOV): 150 × 150 mm; echo time (TE): 108 ms; repetition time (TR): 4500 ms), the axial image of the disc level associated with the greatest neurological compression was selected for measurement. In this study, the L4/5 or L5/S1 intervertebral disc level was selected. Digital measurement of the central canal area outline was performed by tracing the dural cross-sectional area boundaries on the axial MRI at the disc level (Figure 2). Measurements were conducted by a single-blinded musculoskeletal radiologist with more than 10 years of experience, using a previously described method [22]. Each measurement was repeated three times by the same musculoskeletal radiologist to enhance repeatability. The intra-observer intra-class correlation coefficient (ICC) reliability was high (>0.8, 95% CI).

The evaluations of pain (VAS), function (ODI and RM), and morphology (lumbar spine MRI) were conducted within two days of the first and last treatment sessions.

### 2.5. Statistical Analysis—Sample Size Determination

Sample size analysis showed that at least 40 participants were required for a two-sided significance level of 0.05 and an inter-class correlation coefficient of 0.8. Thus, 40 patients were enrolled, considering potential loss to follow-up. Data were analyzed using paired and independent *t*-tests. The significance level was set at *p* < 0.05. All analyses were performed using SPSS software v.22.0 (IBM Corp., Armonk, NY, USA).

## 3. Results

### 3.1. Demographic Characteristics

Patient demographic data are presented in Table 1. The L-LCCT group included 11 females and 9 males, with a mean age of 43.6 ± 15.1 years. The TT group included 12 females and 8 males, with a mean age of 48.0 ± 14.6 years. There were no significant differences between the two groups in terms of sex, age, height, weight, BMI, duration of lumbar pain, initial VAS, ODI score, RM score, or lumbar central canal area (*p* > 0.05). All patients completed the full five-week study period (a total of 15 traction treatment sessions).

### 3.2. Pain, Functional Status, and Morphological Changes

Patients’ pain, functional status, and morphological changes are presented in Table 2. For pain and functional measurements, the L-LCCT group showed superior outcomes compared to the TT group. Results indicated a significant decrease in the VAS pain score for both groups after treatment. However, changes in the ODI and RM were significantly higher in the L-LCCT group, and there were no significant changes in the ODI and RM in the TT group. The change of the central canal area based on lumbar spine MRI was also significantly greater in the L-LCCT group (*p* < 0.05), while there were no significant changes in the central canal area in the TT group after treatment (*p* > 0.05).

Both groups showed significant decreases in pain scores. However, changes in functional scores and central canal area improvements were significantly greater in the L-LCCT group than the TT group (Table 3).

## 4. Discussion

Prolonged periods of pain due to intervertebral disc disease have abnormal neurological mechanisms [23]. Such neurological mechanism changes in intervertebral disc disease patients can cause patients to perform abnormal movements. In addition to neurological abnormalities, patients with HIVD also show asymmetry and atrophy of the spinal muscles on the side of the body with pain. Global muscle spasms and deep-muscle weakness can also result in nonalignment of the vertebrae, leading to pain and adhesion. Thus, neurological and musculoskeletal abnormalities can cause pain and muscular dysfunction in patients with chronic HIVD.

Despite its theoretically favorable effects as a spinal traction device, the clinical usefulness of the TT method in treating lumbar disc disease has been low in clinical settings [24,25,26,27]. This may be due to different treatment guidelines and the individualistic nature of this disease, or to a lack of well-designed studies. In addition, technical limitations such as lordotic curve changes during traction may cause bias in the treatment outcomes. Although the TT method theoretically decreases the lordotic curve in the supine position, the L-LCCT device can also decompress intervertebral disc pressure while maintaining the natural lordotic curve, even in a supine position. In our previous study of cervical lordotic curve-controlled traction (C-LCCT), we demonstrated the positive effects of LCCT (lordotic curve-controlled traction) on the cervical spine [17].

In this study, we also obtained favorable results for the group with L-LCCT compared to TT in terms of pain, function, and morphological outcomes (Table 2). Both groups showed significant decreases in their pain scores after treatment. However, the changes in functional status and the lumbar central canal area were significantly higher in the L-LCCT group than in the TT group.

In addition to pain and functional scores, which could be considered subjective measurements, we also compared morphological changes using lumbar spine MRI. After treatment, the L-LCCT group showed significant widening in the central canal area. However, the TT group did not show any significant differences in the central canal area after treatment. This may be related to the L-LCCT’s ability to maintain or restore the lumbar lordotic curve during spinal traction, thereby reducing the unnecessary muscle guarding to load a sufficient traction force. However, this study has several limitations. First, although we recruited a sufficient sample size, more subjects of different ages will be necessary to generalize our results. As disease status can vary from individual to individual, our results need to be carefully re-evaluated before they can be applied clinically. Age, sex, race, and individual physical factors should also be considered in future studies. Second, vertebral discs can differ in several characteristics including resilience, softness, or severity. In this study, disc disease patients with relatively mild disabilities and low ODI scores were recruited. Regarding geometric status, pain threshold and functional outcome differences could also lead to different outcomes.

Third, although this study recruited patients with more than 3 months of unrelenting intervertebral disc disease, there was no control group without disc disease in this study. As it is possible that disc disease could have resolved spontaneously, a control group with stricter requirements should be included in future studies.

In this study, we obtained immediate responses from patients after traction sessions, and the final outcome measurement was done after completing 15 sessions of traction (approximately 1.5 months). Although this does not reflect the long-term efficacy of traction treatment, we were able to compare the therapeutic effects of the different treatments in a given time. The long-lasting effects of the treatment should be determined in future studies.

Despite these limitations, the newly invented L-LCCT is recommended as an effective treatment method for lumbar intervertebral disc disease. Future studies should be conducted to re-establish traction guidelines such as intensity, interval, and treatment frequency, with the goal of obtaining the best results.

## 5. Conclusions

This study showed that a newly invented traction device that maintained the lumbar lordotic curve was more effective than a traditional traction device in improving pain, functional status, and morphological changes. Thus, this could be an ideal treatment option for patients with lumbar intervertebral disc disease.

## Figures and Tables

**Table 1:** Demographic characteristics of participants.

Variables	All (*n* = 40)	L-LCCT (*n* = 20)	TT (*n* = 20)	*p* Value
Age (years)	45.8 ± 14.7	43.6 ± 15.1	48.0 ± 14.6	0.837
Height (cm)	165.2 ± 8.3	164.6 ± 9.5	165.7 ± 7.0	0.187
Weight (kg)	62.8 ± 11.3	62.8 ± 12.2	62.9 ± 10.8	0.471
BMI	22.9 ± 3.1	23.0 ± 3.2	22.8 ± 3.0	0.954
Duration of lumbar pain (months)	15.5 ± 13.4	12.0 ± 12.1	19.0 ± 14.0	0.226
Initial VAS	6.4 ± 1.2	6.3 ± 1.1	6.5 ± 1.4	0.605
Initial ODI (%)	29.2 ± 12.8	30.7 ± 15.4	27.5 ± 9.5	0.547
Initial RM	6.0 ± 3.6	6.3 ± 3.6	5.6 ± 3.7	0.670
Initial lumbar central canal area (mm^2^)	131.1 ± 48.6	130.2 ± 49.9	131.9 ± 48.8	0.986

All values represent mean ± standard deviation. BMI: body mass index; VAS: visual analogue scale (0 = no pain; 10 = worst pain ever); ODI: Oswestry Disability Index (0 = no disability; 100 = maximum disability possible); RM: Roland–Morris Disability Questionnaire (0 to 24; higher scores represent higher levels of pain-related disability). L-LCCT: the lumbar lordotic curve-controlled traction device, TT: traditional traction.

**Table 2:** Pain, functional, and morphological measurements in the L-LCCT group versus the TT group.

Variables	Before Treatment	After Treatment	*p*-Value
A. L-LCCT group			
VAS	6.3 ± 1.1	3.1 ± 0.8	<0.001 *
ODI (%)	30.7 ± 15.4	20.8 ± 11.6	<0.004 *
RM	6.3 ± 3.6	3.7 ± 2.4	<0.001 *
Central canal area (mm^2^)	130.2 ± 49.9	136.2 ± 49.9	<0.001 *
B. TT group			
VAS	6.5 ± 1.4	4.1 ± 1.6	<0.001 *
ODI (%)	27.5 ± 9.5	25.5 ± 11.6	0.331
RM	5.6 ± 3.7	5.0 ± 4.3	0.305
Central canal area (mm^2^)	131.9 ± 48.8	132.0 ± 48.5	0.988

All values represent mean ± standard deviation. VAS: visual analogue scale (0 = no pain; 10 = worst pain ever); ODI: Oswestry Disability Index (0 = no disability; 100 = maximum disability possible); RM: Roland–Morris Disability Questionnaire (0 to 24; higher scores represent higher levels of pain-related disability); * *p* < 0.05.

**Table 3:** Comparison of changes between L-LCCT and TT groups.

Variables	L-LCCT	TT	T	*p*-Value
VAS	−3.1 ± 1.3	−2.4 ± 1.3	−1.6	0.121
ODI (%)	−7.9 ± 7.6	−2.0 ± 6.9	−2.6	<0.05 *
RM	−2.0 ± 1.5	−0.6 ± 2.1	−2.4	<0.05 *
Central canal area (mm^2^)	6.0 ± 4.8	0.0 ± 6.3	−3.0	<0.05 *

* *p* < 0.05.
